# Pharmacological affinity fingerprints derived from bioactivity data for the identification of designer drugs

**DOI:** 10.1186/s13321-022-00607-6

**Published:** 2022-06-07

**Authors:** Kedan He

**Affiliations:** Physical Sciences, Eastern Connecticut State University, 83 Windham St, Willimantic, CT 06226 USA

**Keywords:** New psychoactive substances, Pharmacological affinity fingerprint, Bioactivity data, Similarity search, Unsupervised clustering, Machine learning

## Abstract

Facing the continuous emergence of new psychoactive substances (NPS) and their threat to public health, more effective methods for NPS prediction and identification are critical. In this study, the pharmacological affinity fingerprints (*Ph-fp*) of NPS compounds were predicted by Random Forest classification models using bioactivity data from the ChEMBL database. The binary *Ph-fp* is the vector consisting of a compound’s activity against a list of molecular targets reported to be responsible for the pharmacological effects of NPS. Their performance in similarity searching and unsupervised clustering was assessed and compared to 2D structure fingerprints Morgan and MACCS (1024-bits ECFP4 and 166-bits SMARTS-based MACCS implementation of RDKit). The performance in retrieving compounds according to their pharmacological categorizations is influenced by the predicted active assay counts in *Ph-fp* and the choice of similarity metric. Overall, the comparative unsupervised clustering analysis suggests the use of a classification model with Morgan fingerprints as input for the construction of *Ph-fp*. This combination gives satisfactory clustering performance based on external and internal clustering validation indices.

**Supplementary Information:**

The online version contains supplementary material available at 10.1186/s13321-022-00607-6.

## Introduction

"Designer drugs" or new psychoactive substances (NPS) are compounds that slightly modify the molecular structure of existing controlled substances to mimic their pharmacological effects and bypass legislation [1, 2]. Terms such as “research chemicals, bath salts, fertilizers, incense, and plant foods” are used to circumvent legislation designed to control the supply and distribution of these substances. According to the United Nations Office on Drugs and Crime (UNODC), 126 countries have reported a total of more than 1047 NPS as of December 2020 [1]. Using a 24/7 web crawler to capture the real number of NPS shows over 4000 unique substances of interest circulating in the online environment, a number roughly four times greater than that reported in known NPS databases [3]. Many countries have used or amended existing legislation, or innovative legal instruments, as a way to address the prevalence of NPS. For example, the Controlled Substances Act, passed in 1986 in the United States, allows any chemical that is “substantially similar” to a Schedule I or II controlled substance to be treated as a Schedule I substance [2]. In the UK, any substance that is not regulated by the Misuse of Drugs Act 1971 falls within the scope of the Psychoactive Substances Act 2016 [4]. However, the ban on any particular NPS or NPS category has led to a rapid substitution in the market. Given that these compounds will now reach users through more clandestine routes and that synthetic drug overdose mortality is increasing across all age groups, races, genders, and ethnicities, new tools and methods must be developed to more effectively address the current problem of NPS abuse [5].

NPS are a heterogeneous group of substances, often classified according to their chemical scaffoldings, based on the observation that structurally similar compounds may have similar biological activities and exhibit similar spectral behavior [6]. Systematic classification of NPS based on pharmacological effects is very challenging because a drug often interacts with many different biological targets. There is no universally agreed method for classifying NPS, and based on their primary mechanism of action and molecular targets, they have generally been grouped into four somewhat overlapping functional categories related to their chemical structure and pharmacological effects: stimulants, cannabinoids, hallucinogens, and depressants [7–9]. It is very likely that some new compounds do not neatly fit into these ‘four-category’ classification and their effects cross these boundaries. The currently used ‘four-category’ classification system groups together compounds with highly varied chemical structures (such as the synthetic cannabinoids), or mechanistically heterogeneous compounds (such as the depressants) in a practical workable system for clinicians, scientists, law enforcement agencies and other interested parties.

Synthetic stimulants currently represent the largest group of NPS that are monitored by the UNODC and EMCDDA [1, 10]. It include cathinones, aminoindanes, benzofurans, phenethylamines, piperazines, and tryptamines, of which synthetic cathinones are by far the largest group and the most studied. Synthetic stimulants exert their stimulatory effects by increasing the concentrations of the monoamine neurotransmitters dopamine (DA), serotonin (5HT), and to a lesser extent, norepinephrine (NE) in the synaptic cleft [11, 12]. There are two distinct mechanisms of synthetic stimulants: stimulation of neurotransmitter release from the cytosolic pool or synaptic vesicles through inhibition of vesicular monoamine transporter-2 and reversal of transporter influx [13]; and inhibition of neurotransmitters uptake from the synaptic cleft through inhibition of the plasma membrane transporters [14–16]. Synthetic cannabinoids were first formally identified and reported to EMCDDA in 2008. Synthetic cannabinoids represent the largest and most structurally diverse class of designer drugs, and some of these compounds are similar to phyto- and endocannabinoids. Synthetic cannabinoids interact primarily with the endocannabinoids systems and its G-protein-coupled cannabinoid receptor type-1 (CB1) and occasionally cannabinoid receptor type-2 (CB2) [17, 18]. The current hypotheses on how synthetic cannabinoids modulate their effects via these receptors and the difference between the observed clinical effects of traditional cannabis and synthetic cannabinoids include biased signaling at cannabinoid receptors or the disruption of mitochondrial homeostasis [19, 20]. Synthetic hallucinogens from the phenethylamine and tryptamine classes, also known as serotonergic psychedelics, interact primarily with cortical serotonin receptors can inhibit the reuptake and increase the release of serotonin, but display heterogeneous profile at several receptors [21–23]. The 5-HT2A receptor agonism plays a key role in mediating the psychedelic effects of both phenethylamine and tryptamine compounds [24], but the concurrent activation of 5-HT1A receptors has been suggested to contribute to the qualitative effects of tryptamine psychedelics and distinguishing them from phenethylamine psychedelics [25]. Affinity for 5-HT2A and 5-HT2C receptors is also reported correlated with the dose that induces psychedelic effects in humans [26]. Most synthetic hallucinogens have been shown to interact with other monoaminergic targets, including adrenergic, dopaminergic, and histaminergic receptors [21, 22, 27–29]. Unlike phenethylamine, many tryptamines interact with monoamine transporters at pharmacologically relevant concentrations [28, 30, 31]. Synthetic depressants are broadly classified into two sub-categories: synthetic benzodiazepines and synthetic opioids. Synthetic benzodiazepines mediate their effects through interactions at gamma-aminobutyric acid-A (GABA-A) receptors, ion channels that consist of different subunit compositions, responding to the inhibitory neurotransmitter GABA [32, 33]. Synthetic opioids are created to bind to the same receptors in the brain as opiates, such as morphine and codeine, and produce similar effects such as euphoria, anxiolysis, feelings of relaxation and drowsiness [34]. Novel fentanyl analogs and other synthetic opioids interact with G protein-coupled opioid receptors as partial to full agonists at $\mu$-, $\delta$-, and $\kappa$-opioid receptor subtypes, with selectivity for the $\mu$-opioid receptor [35–37].

According to the recommendations of the Advisory Council on the Misuse of Drugs (ACMD), the in vitro testing should be used to demonstrate whether a substance is psychoactive [38]. The use of structural similarities to identify compounds with similar biological activities has been the subject of virtual screening (VS) strategies [39]. Two-dimensional (2D) molecular structure fingerprints have been successfully combined with statistical and machine learning methods for predicting target binding and other properties of molecules [40]. However, ligand-based similarity search methods perform poorly when the number of known ligands is insufficient, such as when there are far more unknown NPS compounds than known NPS compounds. For example, synthetic cannabinoids interact less ambiguously with CB1 receptors but containing very structurally diverse molecules. Synthetic cannabinoids demonstrate limited structural similarity to d9-THC are referred to as synthetic cannabinoids due to their pharmacological mechanisms [41]. Therefore, unless specifically included in reference databases they will typically not be detected in conventional drug screening procedures such as urine tests [42]. Activity cliff, on the other hand, is generally defined as a pair of structurally similar compounds with a large difference in potency. 5F-PY-PICA (PubChem CID 129520948) and 5F-PY-PINACA (PubChem CID 125181281) were identified in 2015 and regarded as putative synthetic cannabinoid receptor agonist. However, both compounds exhibited low affinity and efficacy at CB1 and CB2 receptors in vitro, and failed to elicit the in vivo effects potently induced by other synthetic cannabinoids, which cast doubt on their classification as synthetic cannabinoid receptor agonists [43]. Because of the scarcity of studies on the interaction of synthetic cannabinoids with non-cannabinoid targets, potential effects on non-cannabinoid receptors and different signaling pathways that have yet to be identified cannot be ruled out [19, 44].

In contrast to molecular fingerprint, where it reflects compounds’ chemical structure, the so-called bioactivity profile can be used to quantitatively describe compound interactions with the proteome without taking its chemical structure into account [45]. It was demonstrated for compounds that interact with multiple targets that the comparison by their bioactivity profile rather than by their structures can lead to discovery structurally dissimilar compounds eliciting same biological responses [46]. Several studies have reported that using publicly available bioactivity data to construct such bioactivity fingerprints performs better and has a higher hit rate in classification tasks compared to ECFP4 fingerprints [47–49]. Historical screening assays in PubChem were used to create bioactivity profiles for more than 3,000,000 small molecules. This bioactivity fingerprint, termed *PubChem high-throughput screening fingerprints* (PubChem HTSFPs) included 243 different PubChem bioassays. PubChem HTSFPs is used to retrieve hits that are structurally diverse and different from the active compounds retrieved by chemical similarity-based methods [49].

This study aims to investigate the potential of using fingerprints that encode the compound's bioactive profiles when applied to unsupervised classification methods, also known as clustering, for the selection of representative compounds. Given a set of data points ${X}_{i},\dots ,{X}_{n}$ and some notion of similarity ${s}_{ij}>0$ between all pairs of data points ${X}_{i},{X}_{j}$, the intuitive goal of clustering is to divide the data points into several groups (cluster*s*) such that points in the same group are similar and points in different groups are dissimilar to each other. One of the main limitations of the widely used $k$-Means is the need for a priori setting of the number of clusters ($K$). This method is also not recommended in cases where the size of the clusters is very different. On the other hands, the hierarchical clustering take into account the linkage between data points called a dendrogram, which represents an ensemble of clustering models with every possible $K$. Hierarchical clustering approaches require defining a dissimilarity function and a linkage criterion. Agglomerative clustering is initialized by considering every object as a different cluster to create $N$ singleton clusters. Then the closest two objects are combined, leaving $N-1$ clusters. In each step of the algorithm, which pair of clusters is linked is determined by the linkage criteria, which will greatly affect the results. The dendrogram comprising the clustering model can then be “cut” for any number of clusters $2\le K\le N$. Recently, spectral clustering has attracted great interest in the analysis of biological and chemical data [50–53]. If we do not have more information than similarities between data points, a nice way of representing the data is in form of the similarity graph G = (V, E). Each vertex ${v}_{i}$ in this graph represents a data point ${X}_{i}$. Two vertices are connected if the similarity ${s}_{ij}$ between the corresponding data points ${X}_{i}$ and ${X}_{j}$ is positive or larger than a certain threshold, and the edge is weighted by ${s}_{ij}$. The problem of clustering can now to reformulated using the similarity graph: to find a partition of the graph such that the edges between different groups have very low weights (which means that points in different clusters are dissimilar from each other) and the edges within a group have high weights (which means that points within the same cluster are similar to each other). When constructing similarity graphs the goal is to model the local neighborhood relationships between the data points. A reasonable default candidate of the similarity function is the Gaussian similarity function ${s}_{ij}=\mathrm{exp}(-\frac{{\Vert {d}_{ij}\Vert }^{2}}{2{\sigma }^{2}})$ with the Euclidean distance $d({X}_{i},{X}_{j})$. The graph Laplacian matrix is defined as the difference of two matrices as $L=D-W$, where $D$ is the diagonal degree matrix and $W$ is a matrix of positive weights assigned to the graph edges. The eigenvectors of the normalized graph Laplacian then are used as input for a $k$-Means clustering step for final partition [54].

To take advantage of a large amount of bioactivity data in the ChEMBL database, a pharmacological affinity fingerprint (*Ph-fp*) was developed based on Random Forest (RF) classification models. The RF classification model was trained and cross-validated using bioassay data covering a range of molecular targets that are informative for the pharmacological characterization of the NPS. The *Ph-fp* is much shorter in terms of bit-length compared to conventional molecular structural fingerprints. The similarity of the pharmacological profile of compounds can be quantified by a metric similar to the commonly used Tanimoto coefficient. Since *Ph-fp* is defined in a data-driven manner, it can be updated and adapted to the continuous availability of bioassay data in the public domain. An external NPS set was used to compare the performance of *Ph-fp* with Morgan and MACCS fingerprints in two tasks: similarity search and unsupervised clustering. Both clustering algorithms were parameterized and evaluated using internal and external indices. In particular, we have been interested in addressing the question to what extent the data-driven *Ph-fp* can indeed be used to identify compounds based on classical pharmacological categorization, rather than on their biological activity against a single target.

## Methods and materials

### Dataset acquisition and curation

The ChEMBL database, version 29, was used as the data source**.** [55, 56] A range of major neurotransmitter receptors and transporters were selected for the in vivo pharmacological characterization of NPS compounds. [9, 16, 24, 27, 43, 57–60] The biological activity of a compound is quantified by its affinity (given as *Ki*) and/or its potency (given as *IC50*/*EC50*). Bioactivity data for both human and non-human targets were considered. Each distinct molecular target is defined by its unique UniProtKB ID, and each organism/target/activity type combination is referred to as an assay and separate models were built for each assay dataset. ChEMBL bioactivity data were filtered using the following criteria: (1) only single protein target type is considered; (2) human and non-human organisms [Homo sapiens, Rattus norvegicus, Mus musculus] are considered; (3) activity types of only *Ki*, *IC50*, or *EC50*; (4) assay type is “Binding”; (5) activity relationship defined as “ = ”; (6) activity values reported in standard units nM; (7) MW up to 900. The mean standard activity values were calculated when multiple activity records are available. Assays with less than 50 distinct compounds were discarded. Active compounds were defined as those with p*Ki*, p*IC50*, or pEC50 better than or equal to an affinity cutoff value. For each active compound, 4 decoys were randomly sampled from the benchmarking DUD-E (DUD-Enhanced) database [61] to ensure that the dataset for each assay was reasonably sized and suitable for comparing the performance of machine learning classification models, while avoiding the creation of highly unbalanced data sets. The DUD-E [61] decoy compounds were extracted from the ZINC database [62] and filtered based on physicochemical properties. A topological dissimilarity filter was also applied to avoid active compounds in the decoy sets. As an additional step randomly sampled decoys with Tanimoto similarity coefficient larger than 0.9 were removed. The list of assays used to train each model is available from GitHub repository.

The Molecular ACCess System (MACCS) and Morgan fingerprints as implemented in the RDKit toolkit were calculated as the molecular descriptors and used as input feature for the classification model. The substructural key-based fingerprints, MACCS, encodes the absence (0) and presence (1) of predefined chemical features, and is represented by a 166 binary bitstring [63]. MACCS have been shown to be more discriminating than structural key fingerprints using many more features [63, 64]. Morgan is the RDKit implementation of the ECFP4 extended connectivity fingerprint with radius 2 as 1024-bit vector [65]. Extended connectivity fingerprints haven shown the best performance in comparative tests including virtual screening [66], scaffold-hopping [67], and clustering [68].

In addition to the conventional structural fingerprints MACCS and Morgan, a total of 118 0D – 2D molecular descriptors that are immediately available via the RDKit package were selected. 2D descriptors include are: topological (kappa1 – 3, BertzCT, etc.), compositional (number of rings, number of aromatic heterocycles, etc.), electrotopological state (Estate), MolLogP and MolMR (Wildman and Crippen approach), etc. This set of descriptors is referred to as Mol_fp in this study. The full list can be found in the Supporting document in the GitHub repository.

### Model training, validation, and performance evaluation

Random Forest (RF) [69, 70] classification model was constructed using the *ensemble.RandomForestClassifier* module from the Python *scikit-learn* library. The number of decision trees used was set to [20, 60, 100, 140, 180 and the maximum number of features as the total number of features. Ten-fold Nested cross-validation (CV) is used in model training and validation. Each assay dataset was split into training and test sets with 90:10 ratio using the *model_selection.KFold* module of *scikit-learn*. The training set was used for hyperparameter tuning and then the model was validated with the test set. This process was repeated 10 times by selecting a different 10% of the data for validation and by using a different 90% of the data to develop a new model from scratch. The overall performance was then calculated as a mean of classification performances of the 10 separately developed models on different 10% sets of the validation data. The nested CV ensure that the data used to validate the classifier is not part of the data used to train it, which provides almost unbiased performance estimates [71].

The Matthews correlation coefficient (MCC) was utilized to measure and compare the performance of classification models trained in this study [72]:

1 $$MCC = \frac{{{\text{tp}} \cdot {\text{tn}} - {\text{fp}} \cdot {\text{fn}}}}{{\sqrt {({\text{tp}} + {\text{fp}})({\text{tp}} + {\text{fn}})({\text{tn}} + {\text{fp}})({\text{tn}} + {\text{fn}})} }}$$

where **tp**: true positive,**tn**: true negative, **fp**: false positive (Type I error), **fn**: false negative (Type II error). MCC incorporates the imbalance of the dataset and its invariance to the exchange of classes and is therefore considered a balanced measure of the biased data set [73]. Independent of their ratio in the dataset, the classifier must make correct predictions for both negative and positive cases to obtain a high MCC. It ranges in the interval of [− 1, + 1] and reaches the extreme values of –1 and + 1 in the case of complete misclassification and perfect classification, respectively, while MCC = 0 is the expected value of the coin tossing classifier.

### Pharmacological affinity fingerprint (*Ph-fp)* construction

**Fig. 1:**
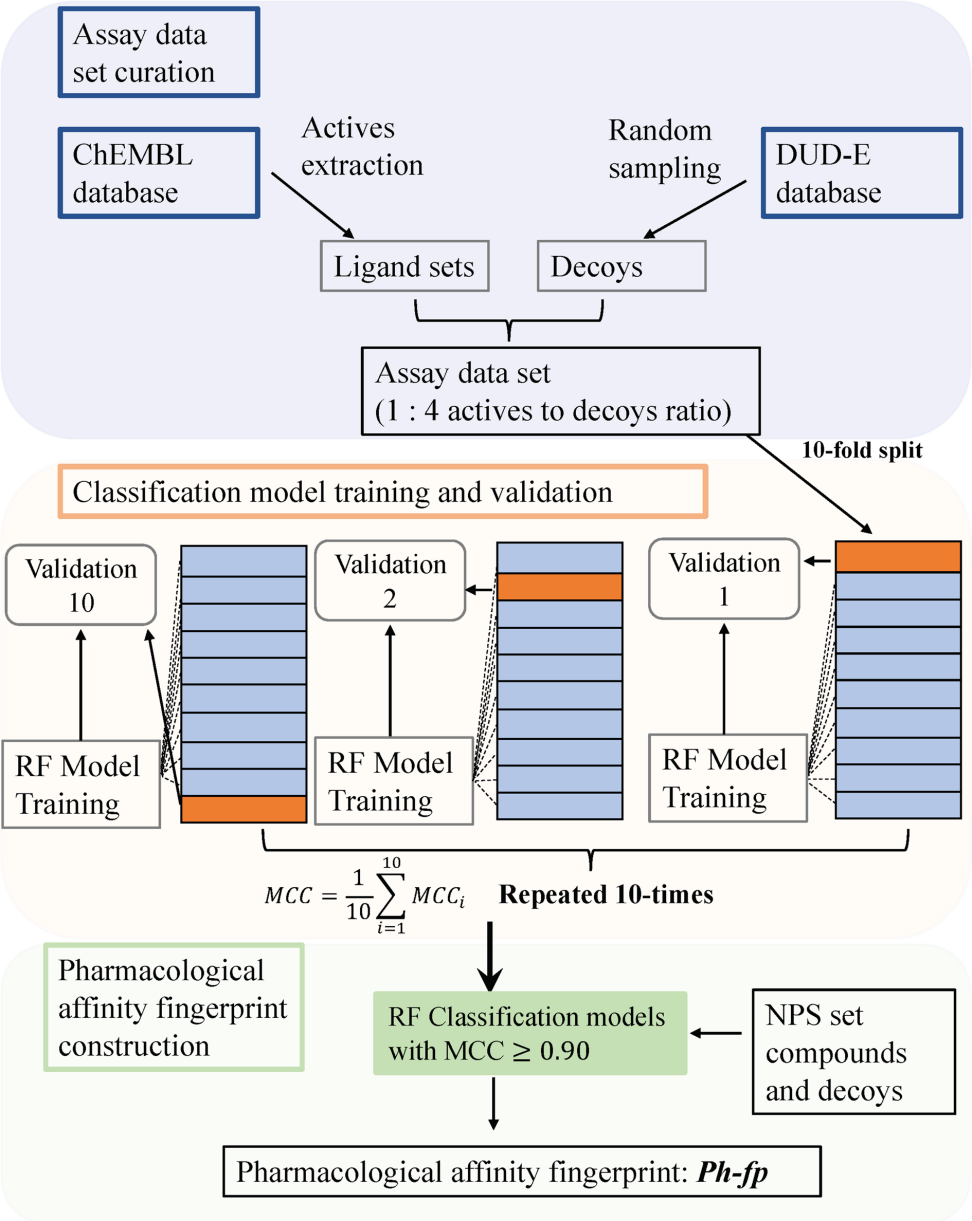
The workflow for the construction of binary pharmacological affinity fingerprint. A total of 132 assay datasets for 70 unique molecular targets were extracted from the ChEMBL 29 database [55, 56]. Each RF classification model was trained with 90% of the data, validated by 10% of the data, and repeated 10 times (tenfold Nested CV). Only models with a mean MCC greater than or equal to 0.90 were included in the *Ph-fp* construction for the NPS set compounds

The *Ph-fp* of a compound is a binary array containing the compound’s activity across the list of target assays predicted by corresponding classification models. Only models with MCC $\ge$ 0.90 were included in the construction of the *Ph-fp*. For each assay, the predictions were repeated 50 times using randomly sampled 90% of the ChEMBL data as training sets. The final prediction is aggregated by majority voting using *sklearn.ensemble.BaggingClassifier*. The workflow for the construction of the *Ph-fp* is shown in Fig. 1.

The NPS set includes 189 compounds collected from the literature and their pharmacological classification was determined based on their in vitro profile data [22, 27, 28, 57–60, 74–77]. Twenty-one natural and synthetic alkaloid and phenylpiperidine opioids and 13 benzodiazepines are classified as depressants; 33 cathinones, 16 phenethylamines, 10 benzofurans, 9 piperidines, 5 aminoindanes are classified as stimulants; 8 THC and derivatives, 14 indoles, and 7 indazole are classified as cannabinoids; 39 phenethylamines (ring-substituted phenethylamines including 2C drugs and their methoxybenzyl [NBOMes] analogues) and 14 tryptamine are classified as serotonergic psychedelics. In this study, the two sub-groups of depressants were separated as individual class because of the unique molecular targets reported in pharmacological studies.

### Pharmacological affinity fingerprint in similarity search performance assessment

**Fig. 2:**
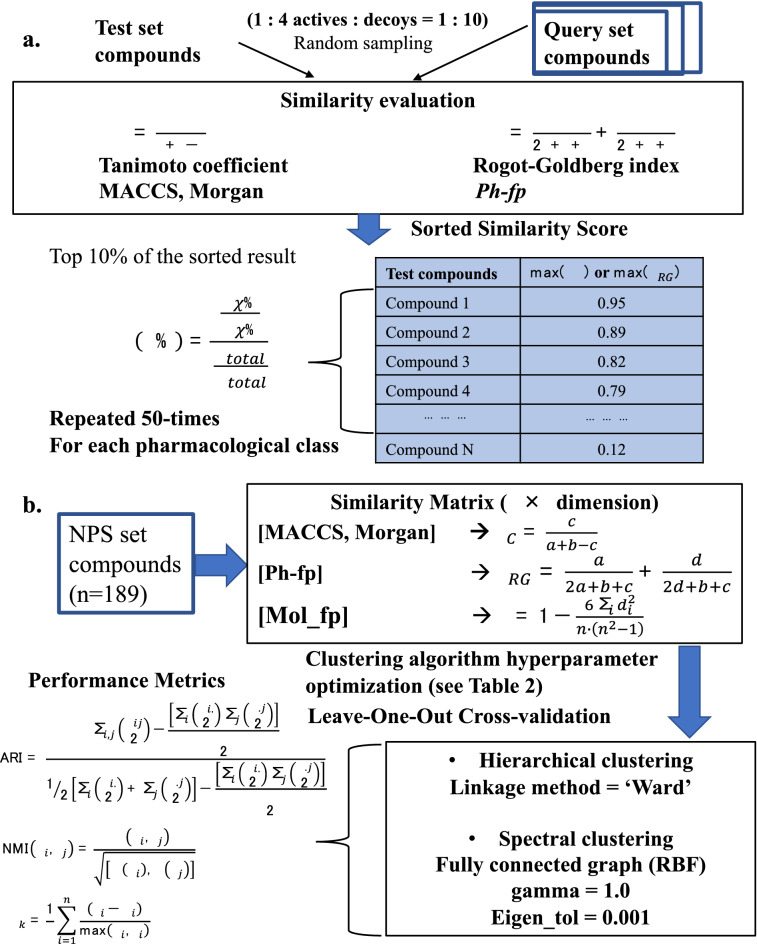
The workflow for the performance evaluation of *Ph-fp* in similarity search and clustering. **a** Similarity search is evaluated using EF10 and AUC. **b** Clustering performance is evaluated using both external (ARI, NMI) and internal (Silhouette score) validation indices

Figure 2a describes the workflow of the performance assessment of *Ph-fp* in similarity search. The similarity of the pharmacological profiles of the molecules described by *Ph-fp* was calculated by the Rogot-Goldberg index [78]:

2 $${S}_{RG}=\frac{a}{2a+b+c}+\frac{d}{2d+b+c}$$

The four basic quantities can be calculated for each pair of fingerprints are:

$a$: the number of 1’s (common “on” bits).

$b$: the number of 1’s present in the first fingerprint but absent in the second.

$c$: the number of 1’s present in the second fingerprint but absent in the first.

$d$: the number of coincident 0’s (common “off” bits).

In comparison to the frequently used Tanimoto coefficient [79] for structural similarity calculated using binary fingerprint, the Rogot-Goldberg index values the information at which targets the compound is inactive as well as which at targets it is active.

The similarity search performance was assessed by two quality metrics, AUC and EF10. AUC is the area under the ROC curve and it quantifies the general ability of a method to discriminate between actives and inactives. AUC equals to the probability that a classifier will rank a randomly chosen positive instance higher than a randomly chosen negative example. Enrichment factor, EF, explicitly measures the early recognition performance. EF is defined as:

3 $$EF\left(\chi \%\right)=\frac{\frac{{P}_{\chi \%}}{{N}_{\chi \%}}}{\frac{{P}_{total}}{{N}_{total}}}$$

where $\chi \%$ is the fraction of the sorted dataset EF is calculated for, ${P}_{\chi \%}$ is the number of actives in this fraction and ${N}_{\chi \%}$ is the number of all molecules in this fraction, whereas ${P}_{total}$ and ${N}_{total}$ are the number of actives and the total number of molecules in the dataset. In this study, EF10 at top 10% ($\chi =0.10$) of the sorted data set was calculated.

The performance of the *Ph-fp* in similarity searching was evaluated using the NPS set and compared with Morgan and MACCS fingerprints. For each experiment, 50 similarity searches were performed for each pharmacological class using different randomly selected test sets where actives were defined as NPS compounds from the pharmacological class of interest. The query set consisted of 10 actives, and 10 decoys randomly selected for each active. The remaining actives of this class and 10 randomly sampled decoys for each active formed the test set to maintain the same 10:1 decoy to actives ratio. The fraction of actives in the test set ($\frac{{P}_{total}}{{N}_{total}}$) is kept constant at 0.091. For each compound in the test set, its similarity to the query compound is calculated, and its nearest neighbor with the highest similarity is retained. The entire test set is then sorted by decreasing similarity and the AUC and EF10 are calculated based on this sorted list (see Table 1).

**Table 1:** NPS set compounds pharmacological categorization and primary molecular target

Pharmacological category [7, 12]	Target(s)	Actives
Stimulants	NET, DAT, SERT	73
Cannabinoids	CB1, CB2	29
Serotonergic psychedelics	5-HT_2A_, 5-HT_2C_	53
Depressant—opioids	$\mu -$ opioid	21
Depressant—benzodiazepines	GABA_A_	13

This NPS dataset is available as a supporting file in GitHub repository: https://github.com/nina23bom/NPS-Pharmacological-profile-fingerprint-prediction-using-ML

### Pharmacological affinity fingerprint in clustering performance assessment

Figure 2b describes the workflow of the performance assessment of *Ph-fp* in unsupervised clustering. The NPS set compounds (n = 189) described by different fingerprints can be transformed into n $\times$ n matrices using appropriate similarity metrics and submitted to hierarchical and spectral clustering algorithms. Structural similarity was calculated using MACCS or Morgan structural fingerprints using Tanimoto coefficients, Spearman’s rank correlation coefficient was used to quantify compound pair similarity using Mol_fp fingerprint, and pharmacological similarity was calculated using *Ph-fp* using Rogot-Goldberg index. In agglomerative hierarchical clustering, four linkage criteria are tested: Ward, complete, weighted average, and single linkage, which measure the proximity between two clusters. In spectral clustering, the n $\times$ n similarity matrix is transformed into a similarity graph in the form of an affinity matrix which is represented by $A$ in different manners: (1) $k$-nearest neighbor graph by connect each point with $k$-nearest neighbors. After connecting the appropriate vertices, the edges are weighted by the similarity of their endpoints. (2) Fully connected graph simply connects all points with positive similarity with each other and weight all edges by ${s}_{ij}$. Compute the first $K$ generalized eigenvectors ${u}_{1},\dots ,{u}_{K}$ of the generalized eigenproblem $Lu=\lambda Du$, where the generalized eigenvectors of $L$ correspond to the eigenvectors of the matrix ${L}_{rw}={D}^{-1}L=I-{D}^{-1}W$. These eigenvectors are used as input in the last $k$-Means step to extract the final partition. The main trick is to change the representation of the abstract data points ${X}_{i}$ to points ${y}_{i}\in {\mathbb{R}}^{K}$. The clustering hyperparameters investigated are listed in Table 2.

**Table 2:** Clustering hyperparameters investigated

Hyperparameters	Parameter	Values explored
*Hierarchical clustering*		
Linkage	Ward	Minimizes the variance of the clusters being merged
	Complete	Maximum distances between all observations of the two sets
	Average	Average of the distances of each observation of the two sets
	Single	Minimum distances between all observations of the two sets
*Spectral clustering*		
Fully connected graph (RBF)	$\gamma$	[1–5]
	eigen_tol	[0.1, 0.01, 0.001,0.0001,0.00001, 0.000001]
$k$-nearest neighbor graph	n_neighbors	[7, 9, 11, 13, 15, 17, 19]
	eigen_tol	[0.1, 0.01, 0.001,0.0001,0.00001, 0.000001]

The *fcluster* and *dendrogram* in *scipy.cluster.hierarchy* package are used for hierarchical clustering, the *SpectralClustering* in *sklearn.cluster* package are used for spectral clusterings

The Leave-one-out cross validation was used in all the clustering analysis and the averaged results of n iterations were reported. Both internal and external indices were used to measure the quality of the clustering partition. The internal indices Silhouette score [80] estimate the quality of a partition by measuring how closely each instance is related to the cluster and how well-separated a cluster is from other clusters given the number of desired clusters *K*. Silhouette score ranges from $-$ 1 to + 1, where + 1 means clusters are well apart from each other and clearly distinguished, $-$ 1 indicates member is assigned to the wrong cluster. On the other hand, external validation indices measure the similarity between the output of the clustering algorithm and the correct partitioning of the dataset [81]. In this study the clustering success defined as correctly identify the Maximum Common Substructure (MCS) based clusters and/or the five pharmacological classes are evaluated using the adjusted Rand-Index (ARI) [82] and the normalized mutual information (NMI) [83]. When two sets of cluster labels have a perfect one-to-one correspondence, the ARI equal to unity. NMI = 0 mean two partitions contain no information about one another, whereases NMI = 1 indicates two partitions contain perfect information about one another. See Supporting document for more detail.

All hierarchical clusterings were generated using the *fcluster* and *dendrogram* in *scipy.cluster.hierarchy* package; spectral clusterings were conducted using the *SpectralClustering* in *sklearn.cluster* package; Silhouette score, ARI, and NMI values were computed using *sklearn.metrics* package.

## Results and discussion

### Data curation and statistics

**Fig. 3:**
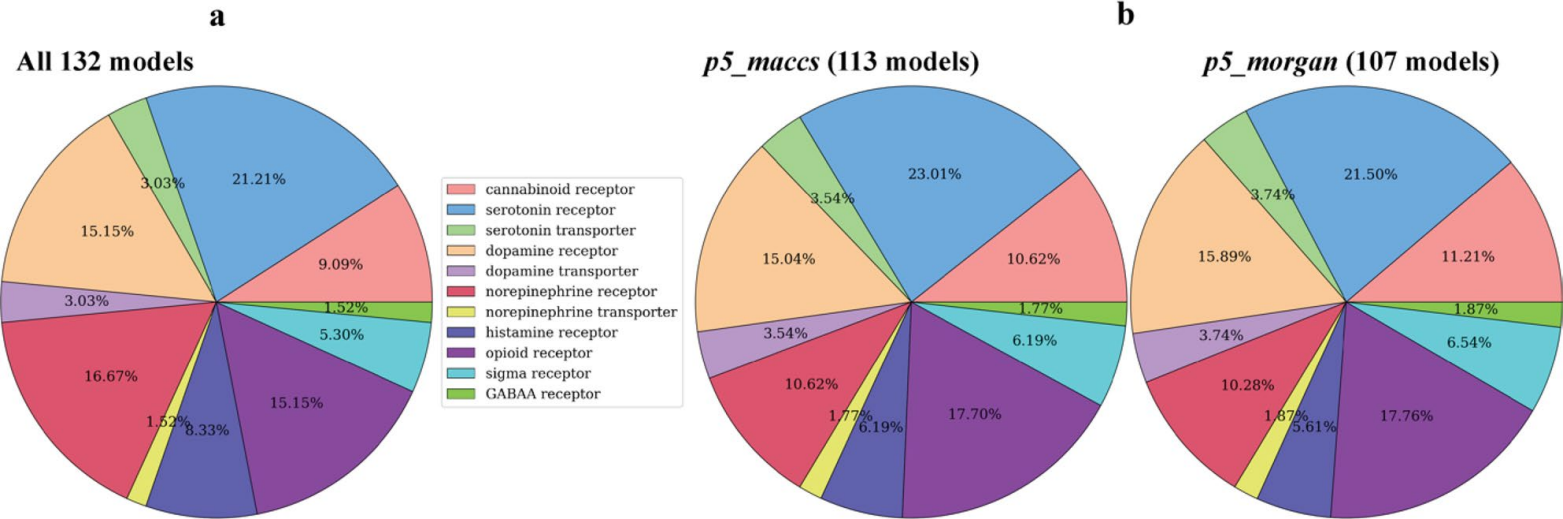
The representation of 11 molecular target classes final models selected for the construction of *Ph-fp*. **a** the total assay datasets trained. **b** final models included in constructing *p5_maccs* and *p5_morgan*. The actives in the assay data are defined as compounds with activity values (pKI, pIC50 and pEC50) greater than or equal to 5

A total of 132 data sets were curated using ChEMBL and DUD-E databases, covering 70 distinct molecular targets from 11 classes. A total of 48 targets were modeled with more than one assay. Three different affinity measurements (pKI, pIC50 and pEC50) with cutoff values greater than or equal to 5 (10 μM), 6 (1 μM) and 7 (10 nM) were used to define the active compound. When a tighter affinity cutoff was applied, fewer assay datasets were used during model training due to fewer compounds labeled as active. Assay datasets with less than 50 unique active compounds were further discarded, resulting in 132, 126, and 116 models being built when using cutoff 5, 6, and 7, respectively. The ratio of decoys to actives is kept at 4:1 for all assay datasets. Hence in average there are 3880 data in each assay dataset using cutoff 5. To construct the binary *Ph-fp* for the NPS set compounds, only the models with MCC $\ge$ 0.90 were included to ensure sufficiently high predictive power. Six versions of the *Ph-fp* were constructed using assay datasets created with different affinity cutoff values and molecules encoded by two molecular descriptors, which are referred in the following text as *p5_maccs*, *p6_maccs*, *p7_maccs*, *p5_morgan*, *p6_morgan*, and *p7_morgan*, their final lengths are listed in Table 3. The comparison of the molecular target classes between the total assay datasets trained and final models included in each *Ph-fp* is shown in Fig. 3 using activity cutoff values greater than or equal to 5, which shows that the distribution are preserved in the final *Ph-fp*. An Excel file listing the target name, UniProtKB, ChEMBL assay ID, target type, activity type, and the number of active compounds using all three affinity cutoffs can be found as Supporting Document in the GitHub repository.

**Table 3:** Number of assay datasets used in the RF classification model and final length of *Ph-fp*

	5 (10 μM)	6 (1 μM)	7 (10 nM)
Total assay sets	132	126	116
Final *Ph-fp* using different molecular descriptors			
MACCS (116 bits)	113	110	102
Morgan (1024 bits)	107	106	104

Three different affinity cutoff values and two molecular descriptors were used in the assay data curation and classification model training, and only models with MCC ≥ 0.90 were included in the final *Ph-fp* construction

### Performance of *Ph-fp* in similarity search

**Fig. 4:**
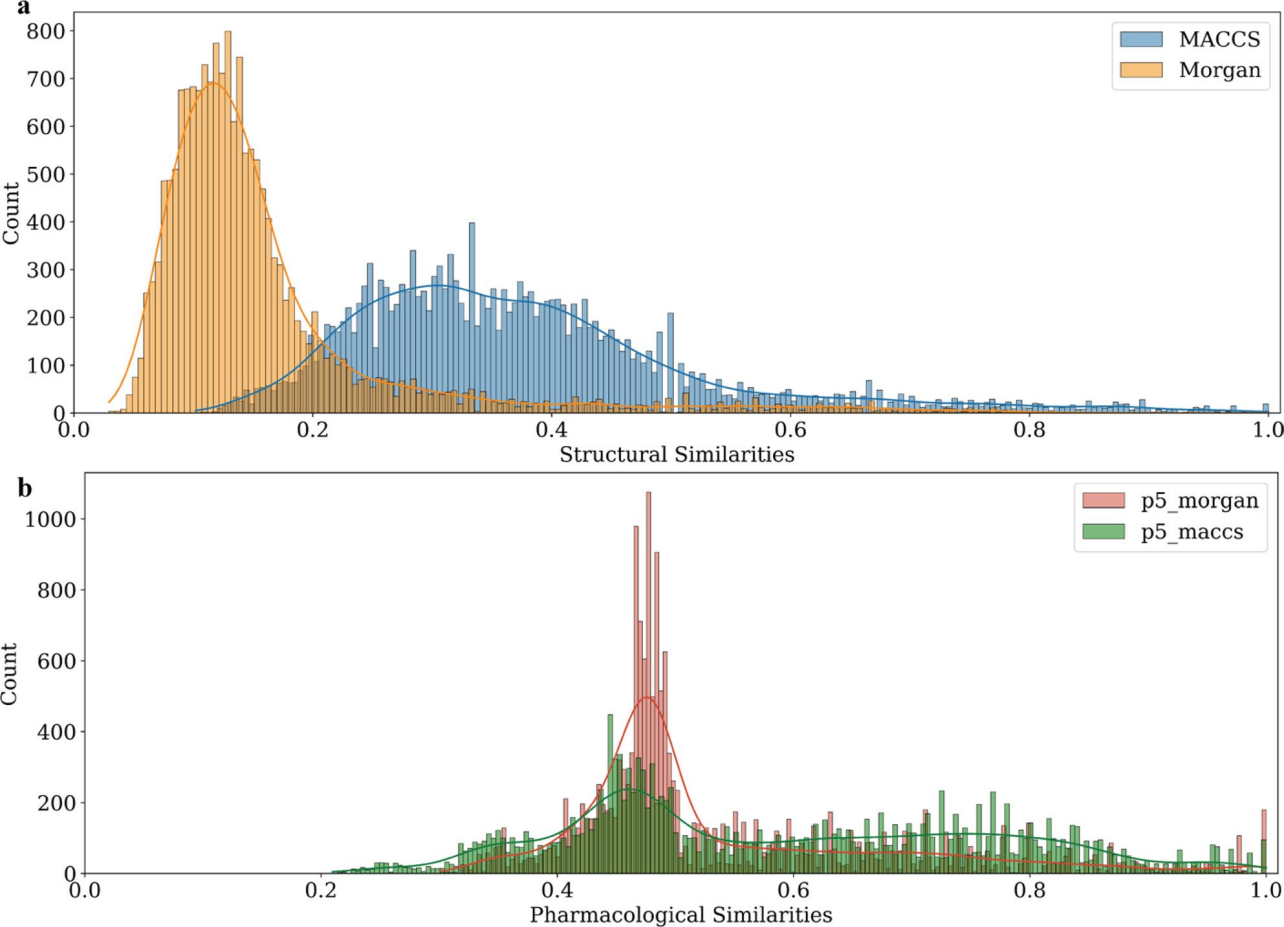
Frequency distribution of pair-wise comparison of NPS set compounds. **a** Structural similarity calculated using Tanimoto coefficient and structural molecular fingerprints, and, **b** Pharmacological similarity calculated using Rogot-Goldberg index and pharmacological affinity fingerprints

**Fig. 5:**
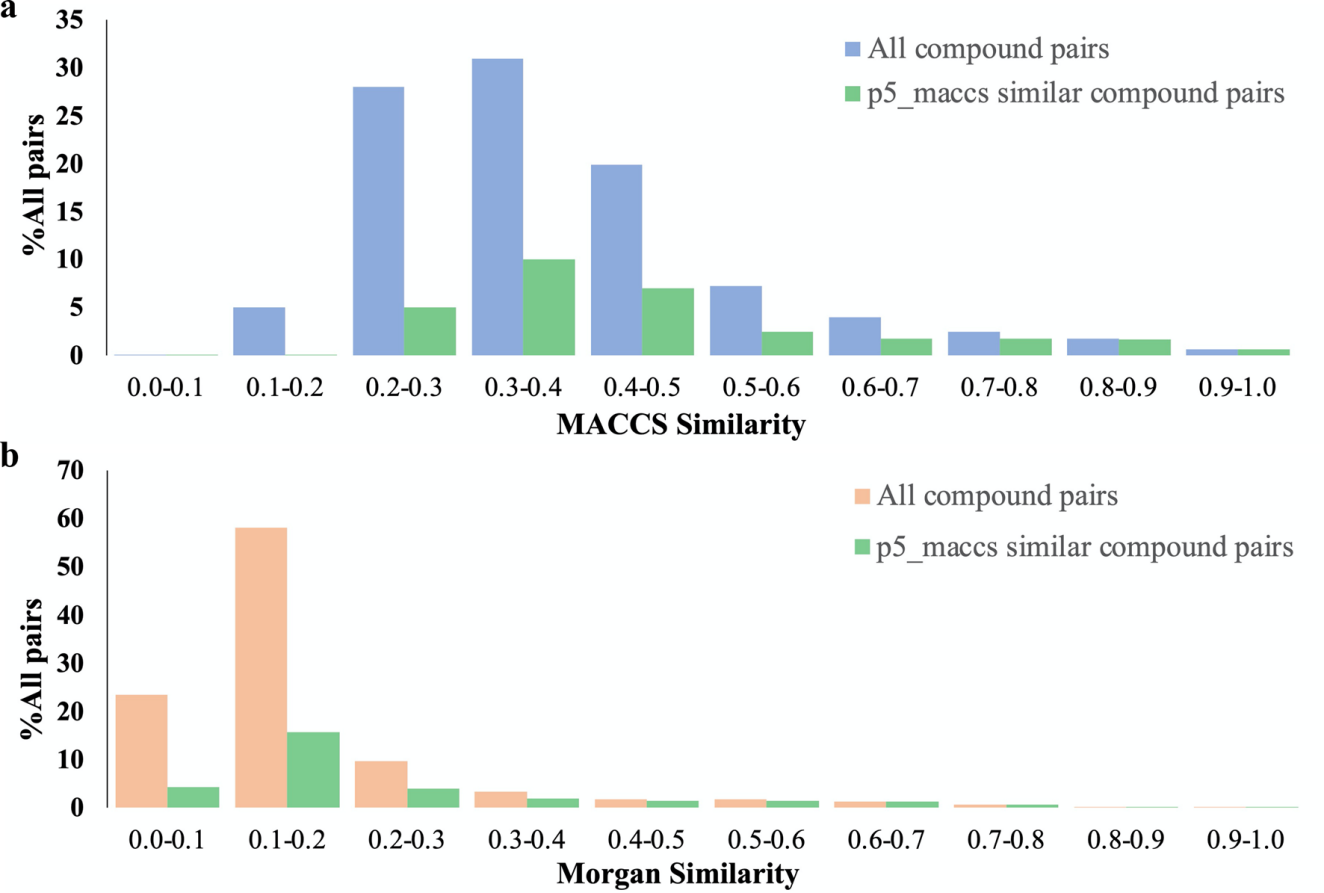
Distribution of the MACCS, Morgan, and *p5_maccs* similarity values between *p5_maccs* similar and *p5_maccs* unsimilar compound pairs

The distribution of pairwise similarity scores of the NPS set compounds was compared by calculating Tanimoto coefficients using structural fingerprints MACCS or Morgan, or Rogot-Goldberg indices using *Ph-fp* fingerprints, as shown in Fig. 4. Figure 4a shows the right-skewed distribution of structural similarity with a medium score of 0.351 and 0.130 using MACCS and Morgan fingerprints, respectively. In contrast, as seen in Fig. 4b, the medium pharmacological similarity is left-shifted and has an asymmetrical long tail with more pairs of compounds on the high-value side. 25% of the compound pairs showed pharmacological similarity scores higher than 0.73 and 0.59 using the *p5_maccs* and *p5_morgan* fingerprints, respectively. In Fig. 5, the level of correspondence between structural and pharmacological similarity of the NPS compounds can be demonstrated. The distribution of MACCS and Morgan similarities for all compound pairs, among which the pharmacologically similar pairs are also shown in the bar chart for comparison. In this analysis, pharmacologically similar compound pairs are defined as having a Rogot-Goldberg index greater than or equal to 0.70 using *p5_maccs*. Only 13.2% of the *p5_maccs* similar pairs have a MACCS similarity above 0.70, while 80.4% of the *p5_maccs* similar pairs have a MACCS similarity of between 0.20 and 0.60. When compared to Morgan, 78.2% of the *p5_maccs* similar pairs have a Morgan similarity below 0.30. A similar distribution pattern can be observed when comparing the structural similarity to other *Ph-fp* similarities.

**Fig. 6:**
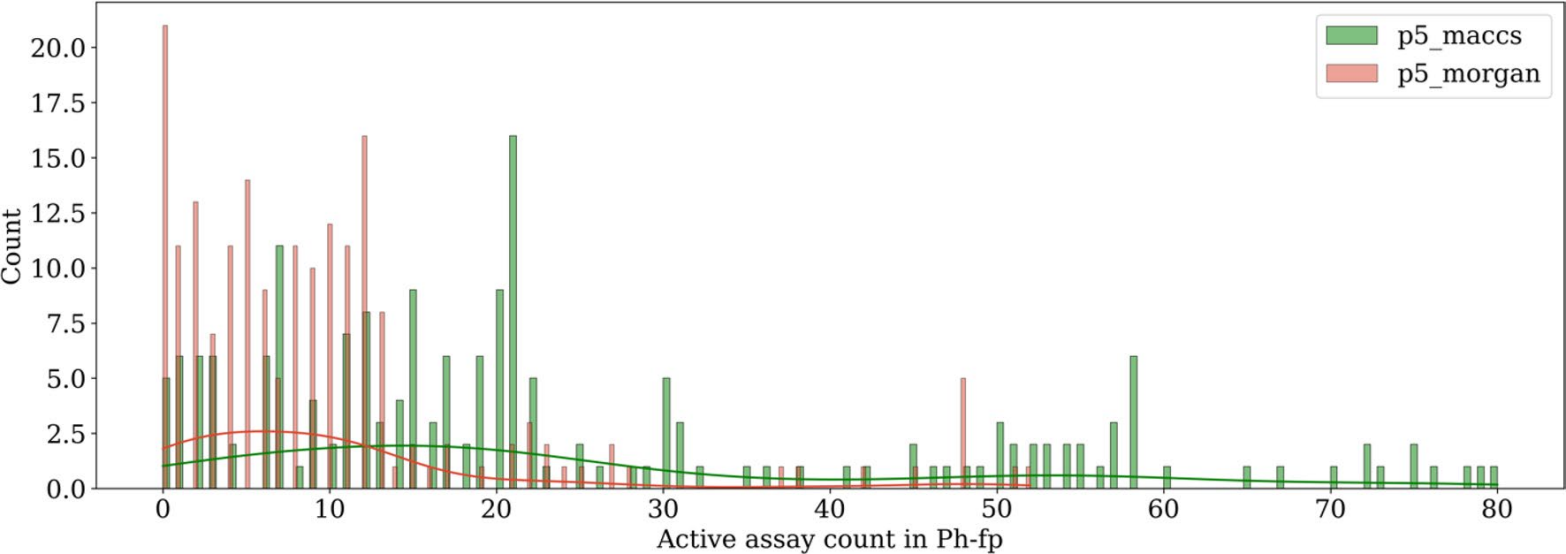
Histogram of the total number of active assay count (“1” bits) of NPS set compounds

In Fig. 4b, the pharmacological similarities are centered at 0.45–0.48 for both *p5_morgan* and *p5_maccs*, although *p5_morgan* regarded more pairs to be median similar, as indicated by the higher peak around this range. Since *p5_morgan* and *p5_maccs* fingerprints are similar in length (107 bits vs. 113 bits) and in assay distribution (see Fig. 2b), this discrepancy stems from the number of active assays predicted by the classification models using different molecular fingerprints. Naturally, the level of similarity between two compounds is affected by the molecular encoding, as well as the similarity metric used. For instance, in the following example: A = (00,000,000) and B = (00,000,000), indicates that both compounds A and B are predicted to be inactive in all eight assays. Using the Tanimoto coefficient, their similarity is calculated to be zero. However, the Rogot-Goldberg index is 0.5. Likewise, A = (10,000,000) and B = (01,000,000) are still considered to be somewhat similar according to the Rogot-Goldberg index of 0.429 since they are both inactive against a total of 6 assays. In Fig. 6, the histogram of the total number of active assay count is plotted for all 189 NPS compounds when described using *p5_maccs* and *p5_morgan*. Upon further inspection, there are a total of 21 NPS compounds predicted to be inactive in all assays according to *p5_morgan*, 19 of which are cathinones (stimulants). In general, classification models using Morgan fingerprints as molecular descriptors predict that NPS set compounds are active in fewer assays and result in more "sparse" (few "1 s" on the bits) binary *Ph-fp* fingerprints. Therefore, more compound pairs were calculated as having a Rogot-Goldberg index of about 0.5 using *p5_morgan*.

The performance comparison of MACCS and Morgen fingerprints in retrieving compounds of the same pharmacological class is given in Table 4 and is separated for each class. To assess the effect of structural diversity on the similarity search for the retrieval of compounds belonging to the same pharmacological class, a similarity threshold was defined as: ${S}_{c}=Z\sigma +y$. Here $y$ is the average and $\sigma$ is the standard deviation of the Tanimoto similarity of the $k$ ($=5$) nearest neighbors of each compound in the pharmacological class. $Z$ is an empirical parameter to control the significant level and set as 0.5. The smaller the ${S}_{c}$, the more structurally diverse the compound set. This similarity threshold value was calculated using MACCS and is listed in Table 4 for each pharmacological class. It shows that the compounds considered as stimulants are the most structurally diverse, while depressants—benzodiazepines are quite similar to each other. The same conclusion is supported by the ${S}_{c}$ calculated using Morgan as well. It is then expected that similarity searches using structural fingerprints to identify compounds in the same pharmacological class should perform better when the structural diversity of the compounds is small. This is confirmed by the results. Overall, Morgan had better performance based on EF10 and AUC, but among all pharmacological classes, stimulants were the most difficult to identify by similarity search using structural fingerprints. The optimal threshold was also calculated from the ROC curve for each pharmacological class similarity search, which is defined as the threshold corresponding to the maximal G-Mean = $\sqrt {{\mathbf{tp(1 - fp)}}}$. The lowest similarity thresholds are required to correctly distinguish stimulus-like compounds compared to other classes.

**Table 4:** Performance comparison of MACCS and Morgan fingerprints in pharmacological class similarity search

	MACCS				Morgan		
	EF10	AUC	Opt_thr	${S}_{c}$	EF10	AUC	Opt_thr
Stimulants	4.15 $\pm$ 0.81	0.67 $\pm$ 0.07	0.72 $\pm$ 0.03	0.26	7.70 $\pm$ 0.78	0.95 $\pm$ 0.03	0.34 $\pm$ 0.02
Cannabinoids	7.61 $\pm$ 0.81	0.95 $\pm$ 0.03	0.76 $\pm$ 0.02	0.30	8.61 $\pm$ 0.98	0.97 $\pm$ 0.07	0.39 $\pm$ 0.05
Serotonergic psychedelics	7.45 $\pm$ 0.86	0.91 $\pm$ 0.06	0.78 $\pm$ 0.03	0.34	8.96 $\pm$ 0.70	0.99 $\pm$ 0.03	0.39 $\pm$ 0.05
D-opioids	8.23 $\pm$ 1.00	0.98 $\pm$ 0.02	0.77 $\pm$ 0.03	0.43	9.26 $\pm$ 0.76	0.99 $\pm$ 0.01	0.44 $\pm$ 0.09
D-benzodiazepines	7.55 $\pm$ 2.13	0.94 $\pm$ 0.06	0.80 $\pm$ 0.06	0.52	10.1 $\pm$ 1.61	0.99 $\pm$ 0.01	0.50 $\pm$ 0.06
Average	7.00	0.89			8.92	0.98	

The data shown is the average of 50 similarity searches for each pharmacological class

Both the query and test sets are composed of 1:10 active to decoy ratio by random sampling

Opt_thr is the optimal threshold defined by the maximal G-Mean = $\sqrt{Sensitivity\times Specificity}$

The similarity search results obtained using *Ph-fp* are summarized in Table 5. The table is divided into two parts, AUC and EF10. For each performance metric, the values in row *i* and column *j* of the table represent the percentage difference between the average *Ph-fp* performance minus the average structural fingerprint performance of each pharmacological class. The last column of the table gives the average AUC and EF10 of each *Ph-fp* across all pharmacological classes. Using MACCS or Morgan as a reference, the best results for each performance metric are shown in italics, and the worst results are underlined. In general, no correspondence was observed between the performance of *Ph-fp* and the structural fingerprints used to construct *Ph-fp*. Although Morgan performed best in retrieving NPS compounds of the same pharmacological class, *Ph-fp* constructed with Morgan performed the worst. This can be explained by the lower total active assay counts as demonstrated in Fig. 6. During the similarity search, NPS compounds are compared not only with each other but also with decoys. Most decoys were predicted to be inactive in all assays and had all "off" bits in their *Ph-fp*. The Rogot-Goldberg index between decoys and NPS compounds with sparse “on” bits is still seen as somehow similar. Therefore, it is challenging to efficiently retrieve NPS compounds of the same pharmacological class that also have sparse *Ph-fp*. One potential solution is to expand the list of assay datasets used in the construction of *Ph-fp*. Another piece of supporting evidence is that *Ph-fp* constructed by using Morgan has the worst performance in identifying the depressant benzodiazepine class compounds. This class of compounds has the lowest degree of structural diversity, however, less than 2% of the assays in *Ph-fp* are representative of molecular targets specific to this class of compounds.

**Table 5:** Performance comparison of *Ph-fp* in pharmacological category similarity search

Fingerprint	MACCS					Morgan					Ave
	*Stimu*	*Canna*	*S-psyche*	*D-opioids*	*D-benzo*	*Stimu*	*Canna*	*S-psyche*	*D-opioids*	*D-benzo*	
AUC											
*p5_maccs*	*46.3%*	0.0%	6.6%	− 1.0%	− 9.6%	*3.2%*	− 2.1%	− 2.0%	− 2.0%	− 14.1%	0.94
*p6_maccs*	44.8%	0.0%	4.4%	− 1.0%	− 1.1%	2.1%	− 2.1%	− 4.0%	− 2.0%	− 6.1%	0.95
*p7_maccs*	28.4%	0.0%	6.6%	− 1.0%	1.1%	− 9.5%	− 2.1%	− 2.0%	− 2.0%	− 4.0%	0.94
*p5_morgan*	17.9%	− 3.2%	0.0%	− 6.1%	− 2.1%	− 16.8%	− 5.2%	− 8.1%	− 7.1%	− 7.1%	0.89
*p6_morgan*	22.4%	2.1%	2.2%	− 4.1%	− 17.0%	− 13.7%	0.0%	− 6.1%	− 5.1%	− 21.2%	0.89
*p7_morgan*	1.5%	− 2.1%	− 1.1%	− 7.1%	− 55.3%	− 28.4%	− 4.1%	− 9.1%	− 8.1%	− 57.6%	0.77
EF10											
*p5_maccs*	*103.4%*	0.8%	12.1%	− 3.2%	− 14.6%	*9.6%*	− 10.9%	− 6.8%	− 13.9%	− 35.8%	7.78
*p6_maccs*	96.1%	− 6.18%	6.4%	− 0.6%	− 3.8%	5.7%	− 17.1%	− 11.5%	− 11.7%	− 27.8%	7.73
*p7_maccs*	46.8%	0.7%	5.8%	0.7%	14.6%	− 20.9%	− 11.0%	− 12.1%	− 10.5%	− 13.9%	7.71
*p5_morgan*	− 31.6%	− 60.3%	− 57.6%	− 57.0%	− 1.9%	− 63.1%	− 64.9%	− 64.7%	− 61.8%	− 26.3%	3.99
*p6_morgan*	26.0%	1.8%	− 3.8%	− 8.0%	− 21.3%	− 32.1%	− 10.0%	− 20.0%	− 18.3%	− 40.9%	6.73
*p7_morgan*	− 29.9%	− 45.5%	− 39.2%	− 44.7%	− 89.3%	− 62.2%	− 51.8%	− 49.4%	− 50.9%	− 91.9%	3.39

*Stimu*  Stimulants, *Canna*  Cannabinoids, *S-psyche*  Serotonergic psychedelics, *D-opioids* Depressant opioids, *D-benzo* Depressant benzodiazepine

The data shown is the average of 50 similarity searches for each pharmacological class using each fingerprint

### Performance of *Ph-fp* in Hierarchical and Spectral Clustering

To calculate the external clustering validation indices ARI and NMI, the externally provided class labels must be used. To reflect the two commonly used categorization systems of NPS compounds, the NPS set is labeled using two sets of external class labels, (1) the five pharmacological classes (Stimulants, Cannabinoids, S-psychedelics, D-opioids, and D-benzodiazepines), and (2) the chemical scaffold classes using Maximum Common Substructure (MCS) based approach. For clarity, *K* = Pharm and *K* = MCS are used in the following text to refer to the two different external class labels assigned to the NPS set. The fully annotated NPS dataset is available on GitHub repository. The MCS similarity is calculated by identifying structural overlap by matching atomic elements and bond types using the *rdFMCS* modules implemented in RDKit [84]. The MCS-based clustering was achieved using hierarchical clustering with Ward linkage. A total of 17 classes were determined as the optimal number of clusters by choosing the maximal Silhouette score as the internal validation of the clustering analysis. See Additional file 1 for more detail. The MCS clustering heatmap in Additional file 1: Fig S1 shows two supergroups. Under the first supergroup, all depressants – benzodiazepines are in cluster 1, where depressants—opioids compounds are split into two clusters (clusters 2 and 9) due to the two main core scaffolds of alkaloid and phenylpiperidine opioids. All cannabinoids are also under this supercluster, with THC based derivatives as one tight cluster and other types of cannabinoids split into several clusters due to the shared indoles, and indazole scaffolds. All serotonergic psychedelic compounds are under the other supercluster and split according to common scaffolds of phenethylamines and tryptamine. Stimulant compounds are distributed in both superclusters due to their structural diversity.

**Fig. 7:**
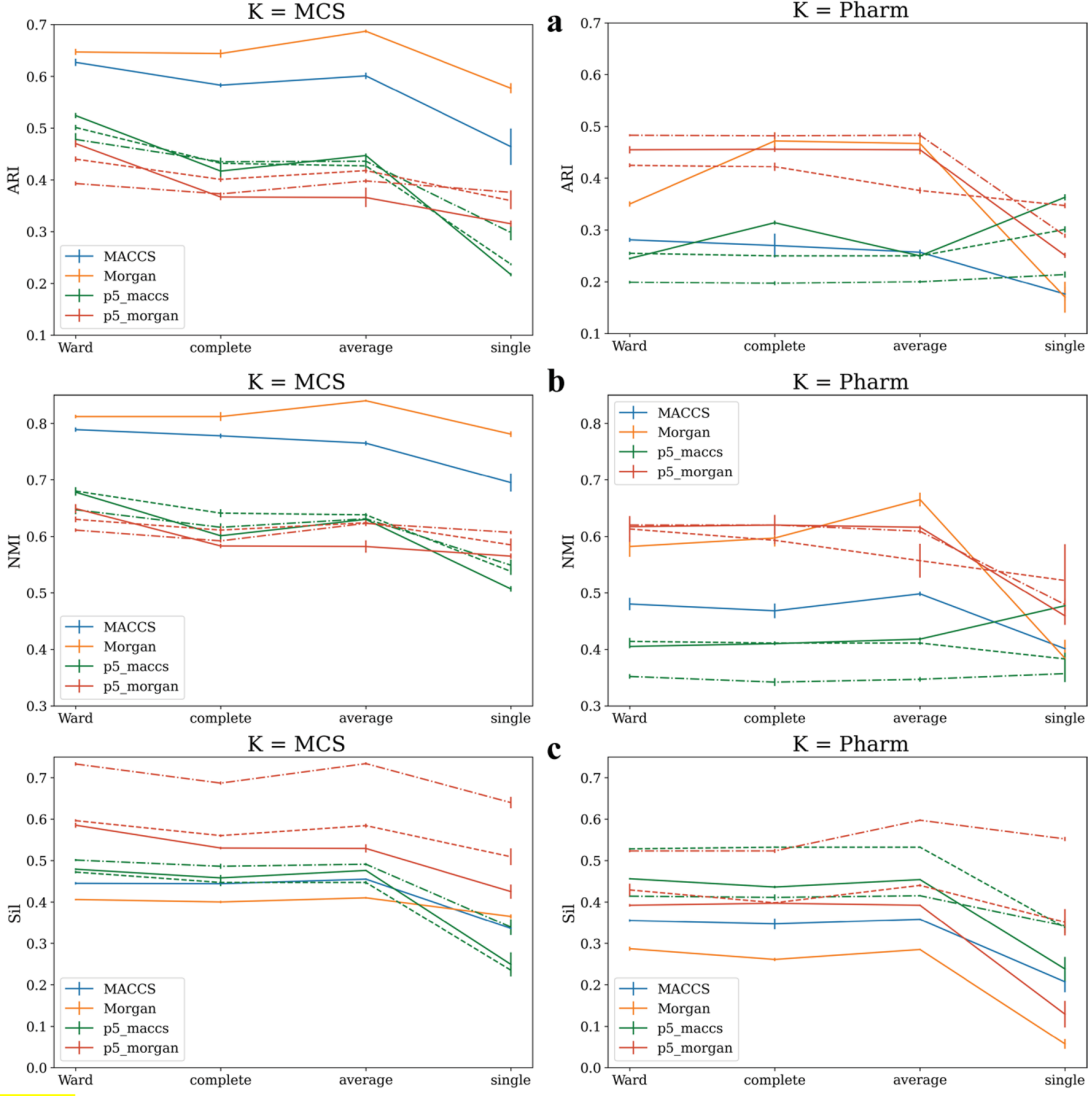
Performance of the hierarchical clustering using different linkage methods. ARI and NMI are calculated by requesting 5 and 17 clusters and comparing them with external *K* = MCS and *K* = Pharm class labels. The dashed lines are *p6_maccs*, *p7_maccs* (green) and *p6_morgan*, *p7_morgan* (red), respectively

The hyperparameters of both clustering algorithms listed in Table 2 were investigated. In Fig. 7, different linkage methods for hierarchical clustering were used and the validation indices calculated using external *K* = MCS and *K* = Pharm class labels are plotted side-by-side for comparison. Figure 7 is divided into three parts, each corresponding to a clustering validation index. Assuming that if *Ph-fp* is indeed describing intrinsic clusters based on the pharmacological characteristics of different classes of NPS compounds, the clustering results using *Ph-fp* fingerprints should be better or at least comparable to structural molecular fingerprints when evaluated using *K* = Pharm class labels, but worse than structural molecular fingerprints when evaluated using *K* = MCS class labels. As expected, MACCS and Morgan performed significantly better than *Ph-fp* in MCS-based cluster discriminations (*K* = MCS) according to ARI and NMI, with Morgan slightly outperforming MACCS. Most interesting, however, was how their performance changed compared to *Ph-fp* when the task was to distinguish clusters based on pharmacological characteristics (*K* = Pharm). In the right-hand panels of Fig. 7, it can be seen that although Morgan gives slightly lower ARI and NMI in this task, the scores still indicate moderate accuracy of the results, while MACCS shows worse performance. Also, the performance deviation of *Ph-fp* depends on how it is generated. Therefore, it is recommended to use Morgan as a molecular descriptor to train the classification models to be used for the construction of *Ph-fp*. Curiously, Morgan had the lowest Silhouette score despite its superior performance in both clustering tasks according to external indices. In contrast, *Ph-fp* clusterings had the highest Silhouette scores, indicating that, on average, the distance between clusters was the largest and the distance within clusters was the smallest when compounds are described by their pharmacological affinity profiles.

The hyperparameter optimization of spectral clustering can be found in the Supporting Document. When using the default parameter gamma = 1 with fully connected graph (affinity = RBF), the same interesting pattern of how the performance “switched” between MACCS and *Ph-fp_morgan* when the task changed from *K* = MCS-based clustering to *K* = Pharm-based clustering (See Additional file 1: Fig S3).

**Fig. 8:**
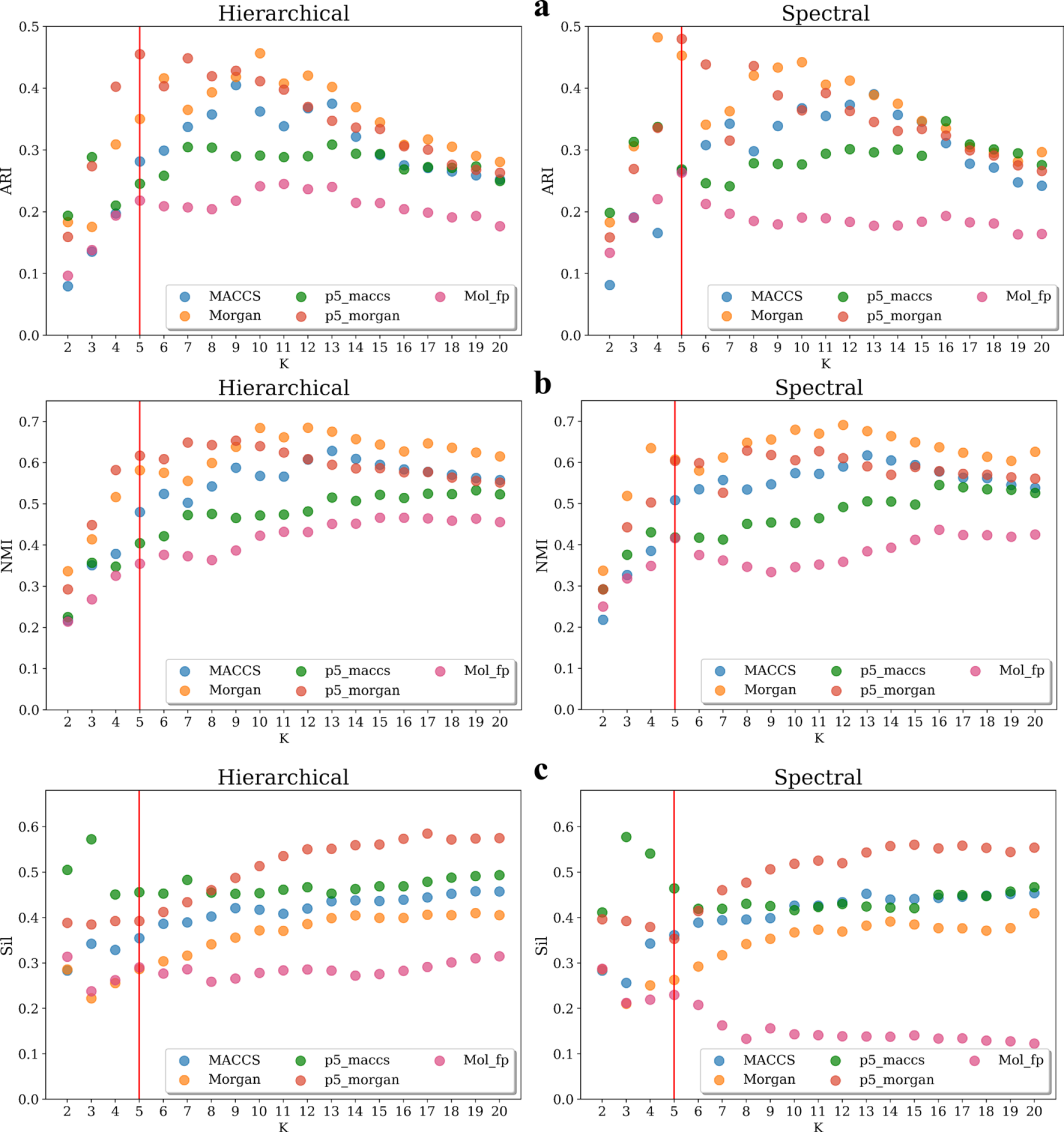
Performance of the algorithms when varying the expected number of clusters K. The ARI, NMI, and Silhouette were calculated by comparing to *K* = Pharm external labels. The red line indicates the five-categories of NPS compounds. The default parameter gamma = 1 was used for spectral clustering, and the Ward linkage method was used for hierarchical clustering

Finally, the performance of the clustering algorithm was verified and compared by setting different values of the requested number of classes *K*. Since the goal is to compare whether the data-driven derived *Ph-fp* provides a comparable or more optimized clustering performance when the objective is to separate NPS compounds based on their pharmacological characteristics, the external class label *K* = Pharm was used to calculate the ARI and NMI external performance indices. The default parameter gamma = 1 was used for spectral clustering, and the Ward linkage method was used for hierarchical clustering. The results obtained using hierarchical and spectral clustering algorithms are plotted side-by-side in two panels in Fig. 8. The red line indicates the five pharmacological classes of NPS compounds expected from this data. Reasonable results can be expected if the clustering algorithm used is appropriate and the description of the compound is appropriate for the clustering task. The results obtained for both algorithms are mostly similar and in agreement with each other for all fingerprints tested. Using all three validation indices, both Morgan and *p5_morgan* fingerprints show satisfactory clustering performance, with *p5_morgan* also showing the highest overall Silhouette score. The highest ARI and NMI were obtained using *p5_morgan* corresponding to *K* = 5. Conversely, setting K < 10 resulted in worse performance for hierarchical clustering using MACCS and Morgan. This result suggests that *Ph-fp* constructed using models trained with Morgan fingerprints as input features is best suited to characterize the pharmacological profile of NPS compounds.

## Conclusion

The previously unseen NPS continue to emerge at an alarming rate posing additional challenges to their accurate and rapid detection. Given the rapid growth in the number of newly synthesized NPS, it is impractical to study all of them in detail. A more economical approach to mitigate the public health threat of NPS is to rapidly screen for active compounds against molecular targets reported to be responsible for the pharmacological effects of NPS. With the increasing availability of HTS data, predictive models can be constructed for each target individually and then subsequently be used to predict the multi-target pharmacological profile of sample compounds. In this study, a data-driven pharmacological affinity fingerprint (*Ph-fp*) was constructed using ChEMBL bioactivity data with Random Forest classification models. The *Ph-fp* consists of biological activities predicted across 132 assay datasets. Two different structural molecular fingerprints, MACCS and Morgan, were used as the input feature in the classification models and assay datasets were further curated using different activity threshold values. The performance of *Ph-fp* in similarity searching and unsupervised clustering was evaluated using a set of NPS compounds. The external class labels for NPS set were assigned based on their five pharmacological categorization (*K* = Pharm) and chemical scaffold categorization (*K* = MCS). In both tasks, the *Ph-fp* was compared to structural molecular fingerprints: 1024 bits long Morgan and 116 bits long MACCS, as well as 118 bits long Mol_fp constructed using 0D-2D molecular descriptors.

The degree of similarity between pairs of compounds is strongly influenced by the encoding of molecular fingerprints, and the use of *Ph-fp* to encode compounds’ predicted pharmacological affinity profiles can provide a complementary perspective when screening for compounds that have the potential to become the next emerging NPS*. Ph-fp* outperformed MACCS in the similarity search in retrieving stimulants with the highest level of compound structural diversity. The poor performance of the *Ph-fp* constructed by the model using Morgan fingerprints as input features demonstrates the importance of expanding the list of assays. Using the Rogot-Goldberg index as the similarity metric overestimated the level of similarity predicted between compounds with fewer "on" bits in their *Ph-fp* and decoys. However, when clustering only the NPS compound set without decoys, *Ph-fp* trained with Morgan can successfully discriminate compounds based on generally accepted pharmacological categorization, with overall superior performance using both external and internal clustering validation indices.

New NPS are emerging at an alarming rate and often without time for adequate experimental determination of their pharmacological profile. In traditional drug testing, if a sample does not match any known substance, it does not yield a positive identification. By definition, designer drugs are made up of chemical combinations that we have not seen before. They almost never match traditional databases. However, a potential strategy is to propose possible structural analogues of popular drugs and synthesize the compounds in the laboratory, and then have their profiles, such as their vibrational or chromatographic spectra, measured and stored in an archive. Then when these drugs become popular in the market, it will shorten the time for positive identification. However, among the endless possible structural analogues, we can further reduce the time cost if we can somehow perform a virtual screening to find the most likely candidate in terms of its potential pharmacological categorization. Thus, when given only the proposed chemical structure, a preliminary virtual screening can be performed using its *Pf-fp* constructed in this data-driven manner using models trained with ChEMBL bioassay data. In summary, data-driven *Ph-fp* is a promising tool for screening potential emerging NPS compounds using public domain bioassay data. Of course, further studies are needed to optimize the list of bioassay data sets used and to further validate the performance of *Pf-fp* using a larger NPS data set. It would be interesting to compare the performance of representative databases constructed using structure-based clustering alone or in combination with the pharmacological space of the NPS in identifying unknown samples.

## Supplementary Information

**Additional file 1:** **Figure S1.** Heatmap of the MCS clustering of NPS set compounds. **Figure S2.** Silhouette analysis for determining optimal clusters *K *of the MCS clustering of NPS set compounds. **Figure S3.** Performance of the spectral clustering by varying gamma parameter. ARI and NMI are calculated by requesting 5 and 17 clusters and comparing them with external *K* = MCS and *K* = Pharm class labels. The dashed lines are *p6_maccs*, *p7_maccs* (green) and *p6_morgan*, *p7_morgan* (red), respectively.

## Data Availability

The datasets and python source code supporting the conclusions of this article are available in the GitHub repository, https://github.com/nina23bom/NPS-Pharmacological-profile-fingerprint-prediction-using-ML.
