# Correction to: Pharmacological affinity fingerprints derived from bioactivity data for the identification of designer drugs

**DOI:** 10.1186/s13321-022-00625-4

**Published:** 2022-07-03

**Authors:** Kedan He

**Affiliations:** Physical Sciences, Eastern Connecticut State University, 83 Windham St, Willimantic, CT 06226 USA

## Correction to: Journal of Cheminformatics (2022) 14:35 https://doi.org/10.1186/s13321-022-00607-6

Following publication of the original article [1], the author identified an error in Fig. 2. The correct figure is given below.

**Fig. 2:**
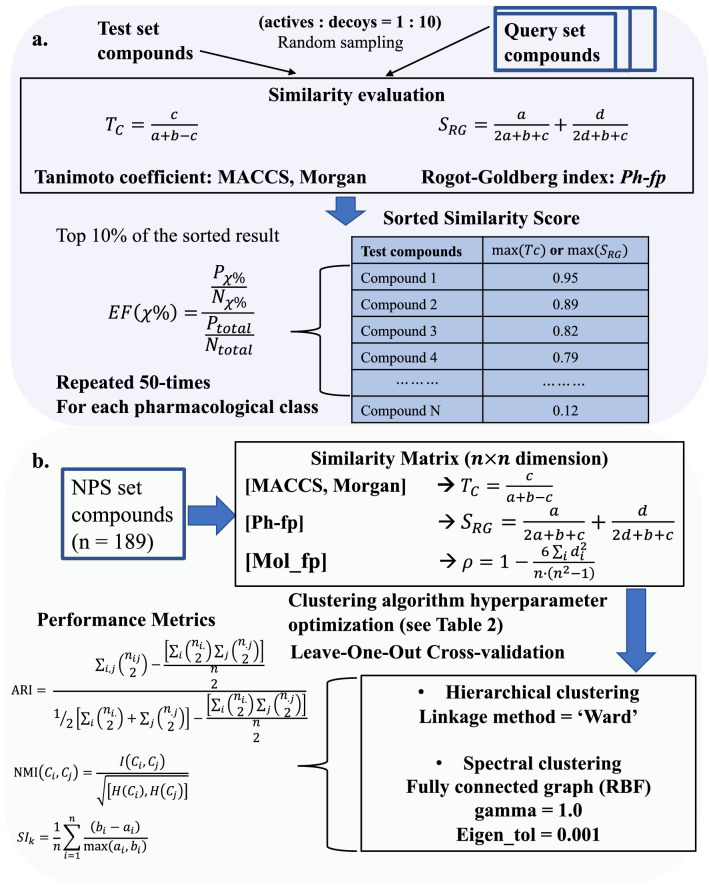
The workflow for the performance evaluation of *Ph-fp* in similarity search and clustering. **a** Similarity search is evaluated using EF10 and AUC. **b** Clustering performance is evaluated using both external (ARI, NMI) and internal (Silhouette score) validation indices

The original article has been corrected.
