# Supplementary material for: Pharmacological affinity fingerprints derived from bioactivity data for the identification of designer drugs
Source: J Cheminform. 2022 Jun 7;14:35. doi: 10.1186/s13321-022-00607-6 (PMC9171973; doi:10.1186/s13321-022-00607-6)
Supplement: Supplementary file 1 — Additional file 1: Figure S1. Heatmap of the MCS clustering of NPS set compounds. Figure S2. Silhouette analysis for determining optimal clusters K of the MCS clustering of NPS set compounds. Figure S3. Performance of the spectral clustering by varying gamma parameter. ARI and NMI are calculated by requesting 5 and 17 clusters and comparing them with external K = MCS and K = Pharm class labels. The dashed lines are p6_maccs, p7_maccs (green) and p6_morgan, p7_morgan (red), respectively. [file 13321_2022_607_MOESM1_ESM.docx]

**Supporting Material**

**Pharmacological affinity fingerprints derived from bioactivity data for the identification of designer drugs**

*Kedan He^*^*

^*^Eastern Connecticut State University, Physical Sciences, 83 Windham St, Willimantic, CT, USA.

^*^Corresponding author: [hek@easternct.edu](mailto:hek@easternct.edu)

**I. GitHub repository**

For datasets and python codes used in this study, please go to the following GitHub repository:

[**https://github.com/nina23bom/NPS-Pharmacological-profile-fingerprint-prediction-using-ML**](https://github.com/nina23bom/NPS-Pharmacological-profile-fingerprint-prediction-using-ML)

## **II. Clustering analysis and validation indices**

**Silhouette analysis** measures the optimal depth of a specified clustering method. It assesses how well each sample $x^{(i)}$ belongs to its assigned cluster $C_{p}$. Each individual Silhouette number is evaluated as:

$$s^{(i)}=\frac{\bar{x}_{C_{q}}^{(i)}-\bar{x}_{C_{p}}^{(i)}}{max(\bar{x}_{C_{q}}^{\left( i \right)},\bar{x}_{C_{p}}^{(i)})} (1)$$

Where $C_{q}$ represents the closest cluster to each $C_{p}$. At each depth on the dendrogram, the average silhouette number is evaluated across all samples and calculated as $\bar{s}=\frac{1}{N}\sum_{i=1}^{N} s^{(i)}$.

External indices measure the similarity between the output of the clustering algorithm and the correct partitioning of the dataset. Different clustering trees were compared with each other using the **adjusted Rand-Index (ARI)**. Let $U=\left\{ u_{1},u_{2},\ldots{,u}_{R} \right\}$ and $V=\left\{ v_{1},v_{2},\ldots,v_{C} \right\}$ represent the external cluster label and that determined by the cluster algorithm, $n_{ij}$ is the number of objects belonging to both subset ${,u}_{R}$ and $v_{j}$, the ARI is calculated:

$$\mathrm{ARI}= \frac{\sum_{i,j} \left( \begin{matrix} n_{ij} \\ 2 \end{matrix} \right)-\frac{\left[ \sum_{i} \left( \begin{matrix} n_{i.} \\ 2 \end{matrix} \right)\sum_{j} \left( \begin{matrix} n_{.j} \\ 2 \end{matrix} \right) \right]}{\begin{matrix} n \\ 2 \end{matrix}}}{\frac{1}{2}\left[ \sum_{i} \left( \begin{matrix} n_{i.} \\ 2 \end{matrix} \right)+\sum_{j} \left( \begin{matrix} n_{.j} \\ 2 \end{matrix} \right) \right]-\frac{\left[ \sum_{i} \left( \begin{matrix} n_{i.} \\ 2 \end{matrix} \right)\sum_{j} \left( \begin{matrix} n_{.j} \\ 2 \end{matrix} \right) \right]}{\begin{matrix} n \\ 2 \end{matrix}}} (2)$$

When two sets of cluster labels have a perfect one-to-one correspondence, the ARI equal to unity. The normalized mutual information (NMI) quantifies the mutual dependence between two random variables based on concepts of information theory:

$$\mathrm{NMI}\left( C_{i},C_{j} \right)=\frac{I\left( C_{i},C_{j} \right)}{\sqrt{\left[ H\left( C_{i} \right),H\left( C_{j} \right) \right]}} (3)$$

where $C_{i}$ and $C_{j}$ are cluster assignments of the points generated from feature subsets of feature selector $i$ and $j$, respectively. Mutual information $I(C_{i},C_{j})$ is given as $H\left( C_{i} \right)-H\left( C_{i} | C_{j} \right)$. $H(C)$ is the Shannon entropy of C, and $H\left( C_{i} | C_{j} \right)$ is the conditional entropy of $C_{i}$ given $C_{j}$. NMI = 0 mean two partitions contain no information about one another, whereases NMI = 1 indicates two partitions contain perfect information about one another.

## **III. Maximum Common Substructure (MCS) based external class label**

The MCS Tanimoto similarity coefficient ($T_{MCS}$) is calculated only on the matched heavy atoms as described:

$$T_{MCS}=\frac{N_{C}}{N_{A}+N_{B}-N_{C}} \left( 4 \right)$$

where $N_{C}$ is the number of matched heavy atoms in MCS of molecule A and B, $N_{A}$ and $N_{B}$ are the number of heavy atoms in molecule A and B, respectively.


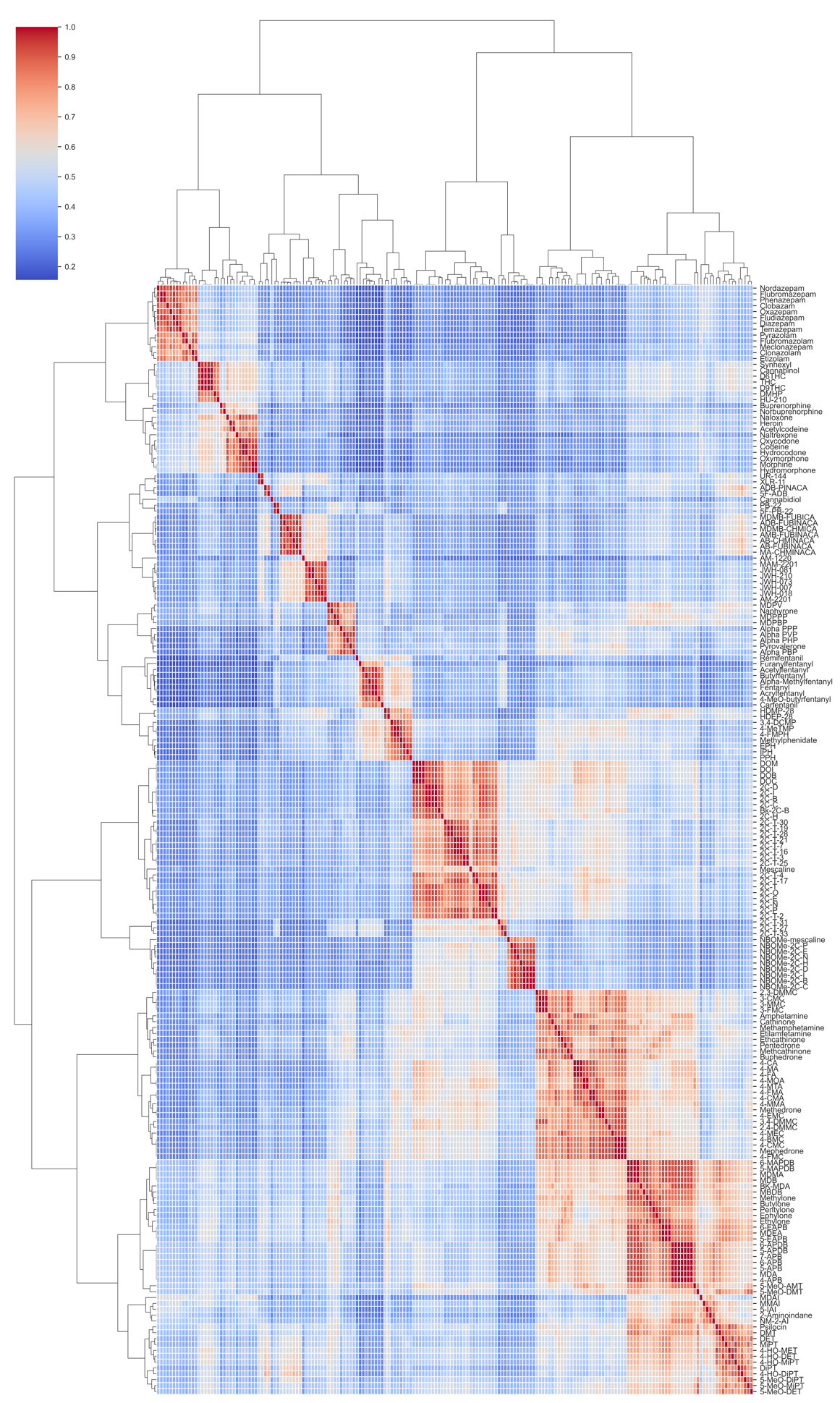


**Fig. S1 Heatmap of the MCS clustering of NPS set compounds**

**
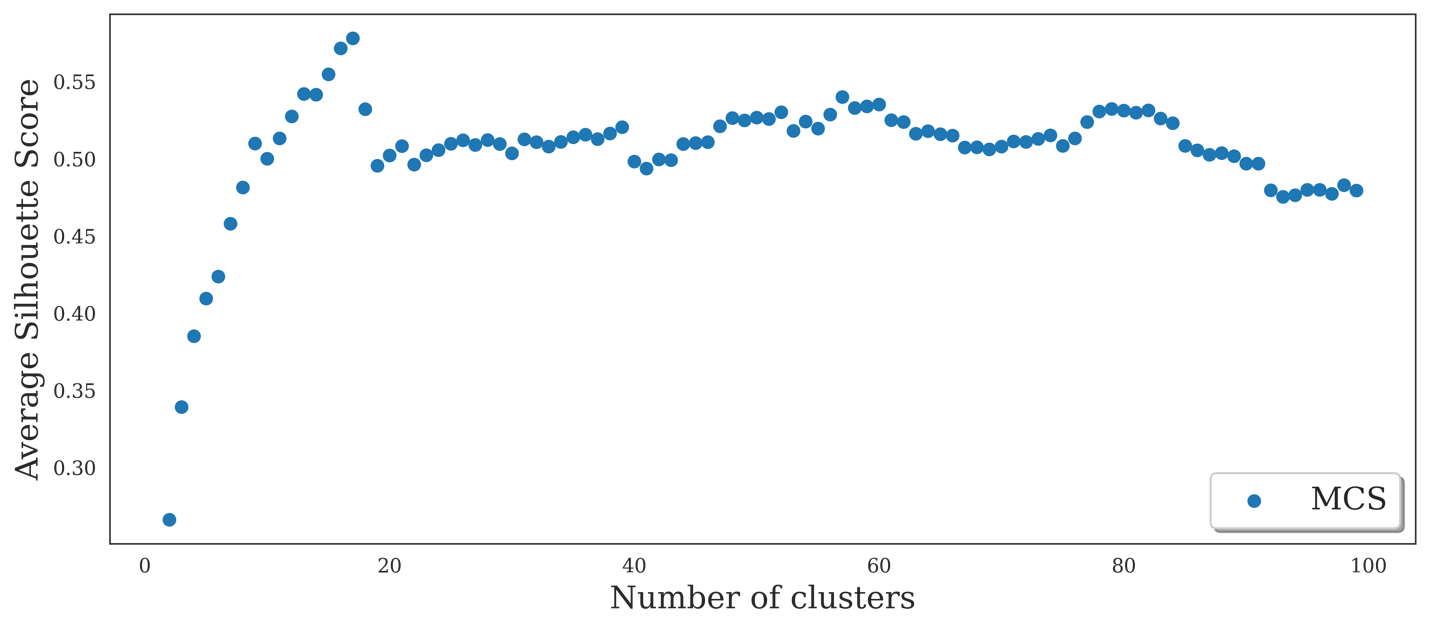
**

**Fig. S2 Silhouette analysis for determining optimal clusters *K* of the MCS clustering of NPS set compounds**

**IV. Spectral Clustering Hyperparameter Optimization**

A grid-search was conducted using two types of similarity graphs ($k$-nearest neighbor graph and fully connected graph), which were evaluated using Silhouette scores. Spectral clustering can be quite sensitive to changes in the similarity graph and the choice of its parameter. In general, if the similarity graph contains more connected components than the number of clusters we ask the algorithm to detect, then spectral clustering will trivially return connected components as clusters. The fully connected similarity graph (affinity = RBF) was used for the final clustering performance assessment using spectral clustering algorithm. The Gaussian similarity function is scaled by the parameter gamma (in *Scikit-learn* implementation), which affects the number of corresponding $k$-nearest neighbor for each data point. As Luxburg points out,^1^ for most data points, the set of neighbors with a similarity significantly greater than 0 should be "not too small and not too large". The performance comparison of the two clustering tasks K = Pharm and K = MCS is shown side-by-side in Fig S3.


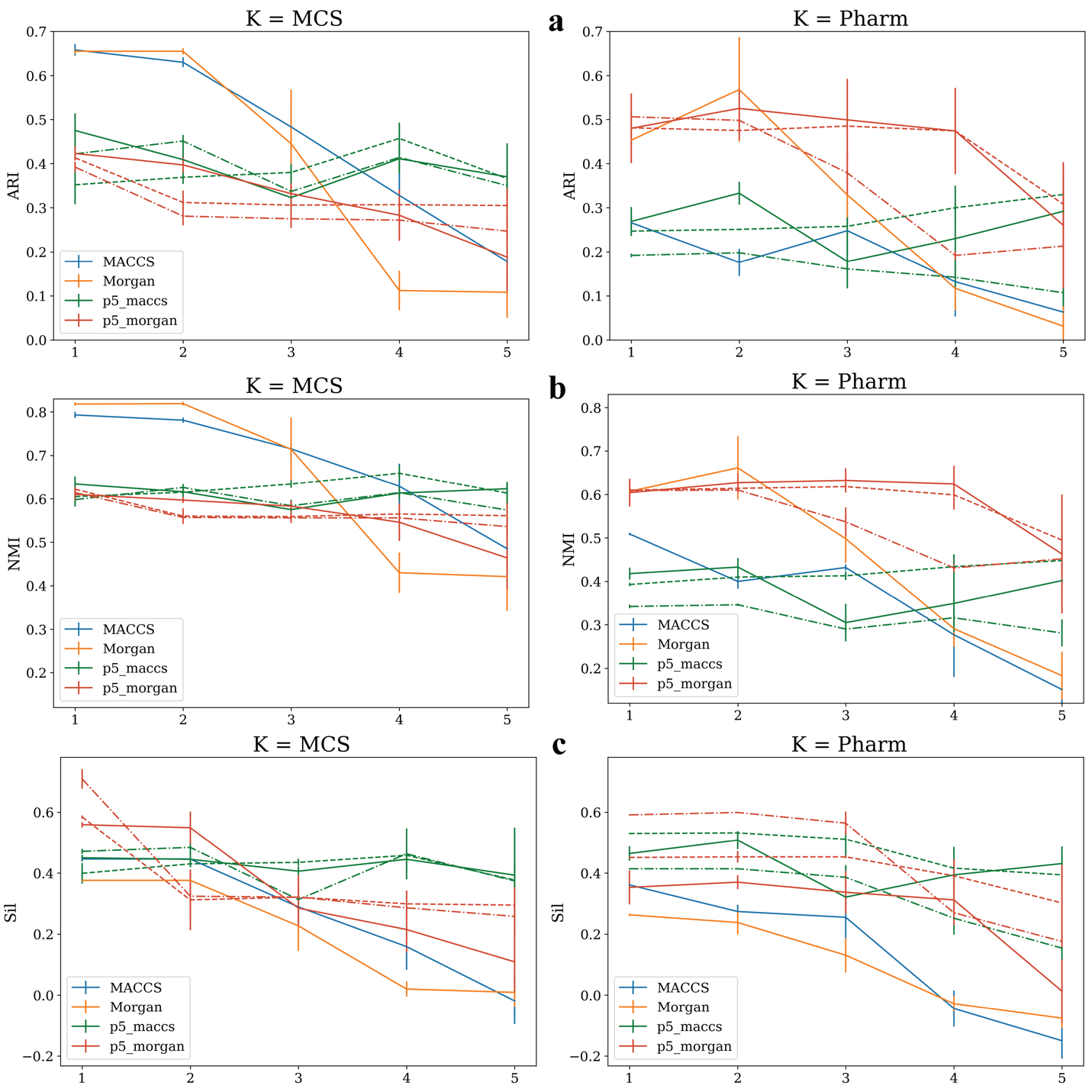


**Fig. S3** **Performance of the spectral clustering by varying gamma parameter.**

ARI and NMI are calculated by requesting 5 and 17 clusters and comparing them with external *K* = MCS and *K* = Pharm class labels. The dashed lines are *p6_maccs*, *p7_maccs* (green) and *p6_morgan*, *p7_morgan* (red), respectively

**Reference**

1. von Luxburg, U. A tutorial on spectral clustering. *Statist. Comput.* **2007,** *17* (4), 395-416.
